# A Pilot Study of Breast Cancer Histopathological Image Classification Using Google Teachable Machine: A No-Code Artificial Intelligence Approach

**DOI:** 10.7759/cureus.87301

**Published:** 2025-07-04

**Authors:** Namit Singla, Abhra Ghosh, Manthan Dhingra, Upajna Pal, Arkajit Dasgupta, Arpan Ghosh, Tae-Yong Kuc, Krisha Singla

**Affiliations:** 1 Medicine, Dayanand Medical College and Hospital, Ludhiana, IND; 2 Biochemistry, Mata Gujri Memorial Medical College and Lions Seva Kendra Hospital, Kishanganj, IND; 3 Cardiology, Guru Nanak Dev Hospital (Associated with Government Medical College, Amritsar), Amritsar, IND; 4 Pathology, Dr. B. C. Roy Post Graduate Institute of Paediatric Sciences, Kolkata, IND; 5 Biochemistry, Teerthanker Mahaveer Medical College and Research Center, Moradabad, IND; 6 Electrical and Computer Engineering, Sungkyunkwan University, Suwon, KOR; 7 Electronic and Electrical Engineering, Sungkyunkwan University, Suwon, KOR; 8 Medicine, Christian Medical College and Hospital, Ludhiana, IND

**Keywords:** ai applications in healthcare, ai in healthcare, artificial intelligence, breast cancer histopathology, digital pathology, machine learning, no-code ai platforms

## Abstract

Introduction

Breast cancer remains a major global cause of cancer-related mortality, where histopathology serves as the diagnostic cornerstone. However, inter-observer variability and increasing diagnostic workload necessitate innovative solutions. This pilot study assesses the feasibility and diagnostic performance of a no-code, browser-based artificial intelligence platform, Google Teachable Machine (GTM; Google Creative Lab, New York, NY, USA), for classifying breast histopathology images into clinically relevant categories.

Methods

A total of 380 hematoxylin and eosin-stained images, equally distributed among four diagnostic categories (normal, benign, in situ carcinoma, invasive carcinoma), were sourced from an open-access repository. The GTM model was trained with 85% of the data (50 epochs; batch size 16; learning rate 0.0001), and externally validated on 39 independent images. Performance metrics included accuracy, precision, recall, and F1-score.

Results

The model achieved an internal validation accuracy of 88.3%, with per-class accuracies of 87% for Normal, 93% for Benign, 87% for In Situ Carcinoma, and 87% for Invasive Carcinoma. On external validation using 39 independent images, the model demonstrated an overall accuracy of 76.9%, with a macro-averaged F1-score of 0.77 and a weighted-averaged F1-score of 0.77. Class-wise external performance metrics included precision, recall, and F1-scores of 1.00, 0.70, and 0.82 for Normal; 0.67, 0.80, and 0.73 for Benign; 0.67, 1.00, and 0.80 for In Situ Carcinoma; and 1.00, 0.56, and 0.71 for Invasive Carcinoma, respectively. The model exhibited high precision across most classes but demonstrated reduced recall for invasive carcinoma, reflecting challenges in distinguishing invasive from non-invasive lesions within the constraints of a limited dataset.

Conclusion

GTM demonstrated preliminary feasibility for multi-class breast histopathology classification using small datasets without coding expertise. While performance was encouraging, particularly for normal and in situ categories, limitations such as reduced invasive carcinoma sensitivity and small sample size underscore the need for larger datasets, advanced architectures, and explainable AI methods to enhance clinical applicability.

## Introduction

Breast cancer represents the most commonly diagnosed malignancy in women worldwide and remains a leading cause of cancer-related mortality. According to GLOBOCAN 2020 data, approximately 2.3 million women were diagnosed globally, resulting in nearly 685,000 deaths, reflecting the significant global burden of this disease [1,2]. Timely and accurate diagnosis plays a crucial role in guiding appropriate therapeutic interventions and improving patient survival outcomes [3].

Histopathological evaluation remains the gold standard for diagnosing breast cancer, offering critical insights into tumor type, grade, and prognostic factors essential for clinical management [4,5]. However, histopathology inherently carries challenges related to subjectivity. Key histological features such as mitotic count, nuclear pleomorphism, and tubule formation, which form the basis of widely used grading systems like the Nottingham Grading System, are susceptible to considerable inter- and intra-observer variability [6-9]. This variability contributes to diagnostic inconsistencies and limits reproducibility. Notably, up to 50% of cases fall into Grade 2, a biologically heterogeneous group with variable clinical behavior [9]. In particular, manual mitotic figure counting - while prognostically valuable - remains labor-intensive and variable, often requiring five to 10 minutes per case [10].

Advancements in artificial intelligence (AI) and machine learning (ML) have introduced powerful tools capable of improving diagnostic consistency, efficiency, and accuracy in histopathology [11]. Deep learning (DL), specifically through convolutional neural networks (CNNs), enables automatic extraction of complex histological features without the need for explicit feature engineering [12,13]. Existing AI-based systems have demonstrated promising results in mitotic figure detection, nuclear pleomorphism assessment, tumor subtype classification, and metastatic lesion detection from digitized hematoxylin and eosin (H&E)-stained whole-slide images [14]. The integration of AI into digital pathology holds potential to support pathologists, reduce variability, and standardize diagnoses across institutions [15-19].

The rapid adoption of whole-slide imaging (WSI) technologies has further facilitated AI integration by providing high-resolution, digitized pathology datasets [20,21]. In addition to improving diagnostic workflows, AI-powered image analysis may offer valuable prognostic and predictive information, supporting patient stratification and personalized therapeutic decision-making [22,23]. For smooth integration of AI into routine diagnostic workflows, several prerequisites must be met, including standardized digital pathology infrastructure, validated AI algorithms with regulatory approval, integration of AI tools into existing laboratory information systems, pathologist training in AI-assisted interpretation, and robust quality assurance protocols to ensure safe and reliable deployment [21,23].

Despite these advancements, widespread implementation of AI in pathology remains hindered by several practical barriers. Developing AI models often requires substantial programming expertise, high-performance computing resources, and access to large annotated datasets [24,25]. These factors limit adoption, particularly in resource-constrained settings.

To address these challenges, accessible no-code platforms such as Google Teachable Machine (GTM; Google Creative Lab, New York, NY, USA) have emerged, allowing users to build image classification models without requiring coding or advanced computational infrastructure [13]. While GTM has shown feasibility in several image classification tasks, its application to multi-class breast histopathology remains underexplored. Furthermore, performance on smaller datasets, its generalizability across independent samples, and limitations in model transparency require systematic evaluation.

This study aims to assess the feasibility and diagnostic performance of a Google Teachable Machine-based model in classifying breast histopathology images into four clinically relevant categories: normal, benign, in situ carcinoma, and invasive carcinoma. Both internal validation and independent external testing were performed to evaluate the model’s robustness, while recognizing the unique accessibility that no-code platforms offer for democratizing AI deployment in low-resource environments where expert pathology services may be limited. Compared to other no-code or AutoML tools such as Lobe.ai and Microsoft Custom Vision, Google Teachable Machine offers a particularly user-friendly interface with minimal setup requirements and seamless browser-based deployment, making it especially suited for educational, pilot, and feasibility studies. While these platforms share similar strengths in accessibility, GTM distinguishes itself through its simplicity and ease of use, as it does not require cloud accounts, subscriptions, or software installations, which may be advantageous in low-resource settings.

## Materials and methods

Dataset collection and classification

This study utilized a curated dataset of H&E-stained breast histopathology images obtained from an open-access repository hosted on Kaggle (https://www.kaggle.com/), made available under a CC0: Public Domain license, which permits unrestricted reuse, redistribution, and adaptation [26]. The dataset consisted of a total of 400 images, equally distributed across four histological categories: Normal (n=100), Benign (n=100), In Situ Carcinoma (n=100), and Invasive Carcinoma (n=100). The images represent regions of interest extracted from whole-slide images and include diverse morphologic features within each class.

All images had been previously annotated based on histopathological diagnosis provided by the original dataset source [26]. To minimize misclassification bias, these annotations were independently reviewed and verified by two experienced pathologists. During review, it was found that the quality of five images in the normal category was unsatisfactory. To maintain class balance, 95 images from each category were selected for use in the model's development. As the dataset contained no patient-identifiable information, institutional ethical approval was not required.

Preprocessing and model development using Google Teachable Machine

The entire dataset was uploaded into GTM [27], a web-based no-code machine learning platform built on TensorFlow.js. GTM automatically performs several preprocessing steps before model training, including resizing images to 224 × 224 pixels, normalizing pixel values between 0 and 1, and randomly shuffling the dataset to ensure balanced representation across training batches.

The dataset was organized into four separate class folders corresponding to the diagnostic categories. GTM automatically allocated 85% of the images for training and 15% for internal validation. This yielded approximately 81 images per class for training and 14 images per class for internal validation. No additional manual preprocessing, such as color normalization, augmentation, or class balancing, was applied during this phase.

**Figure 1:**
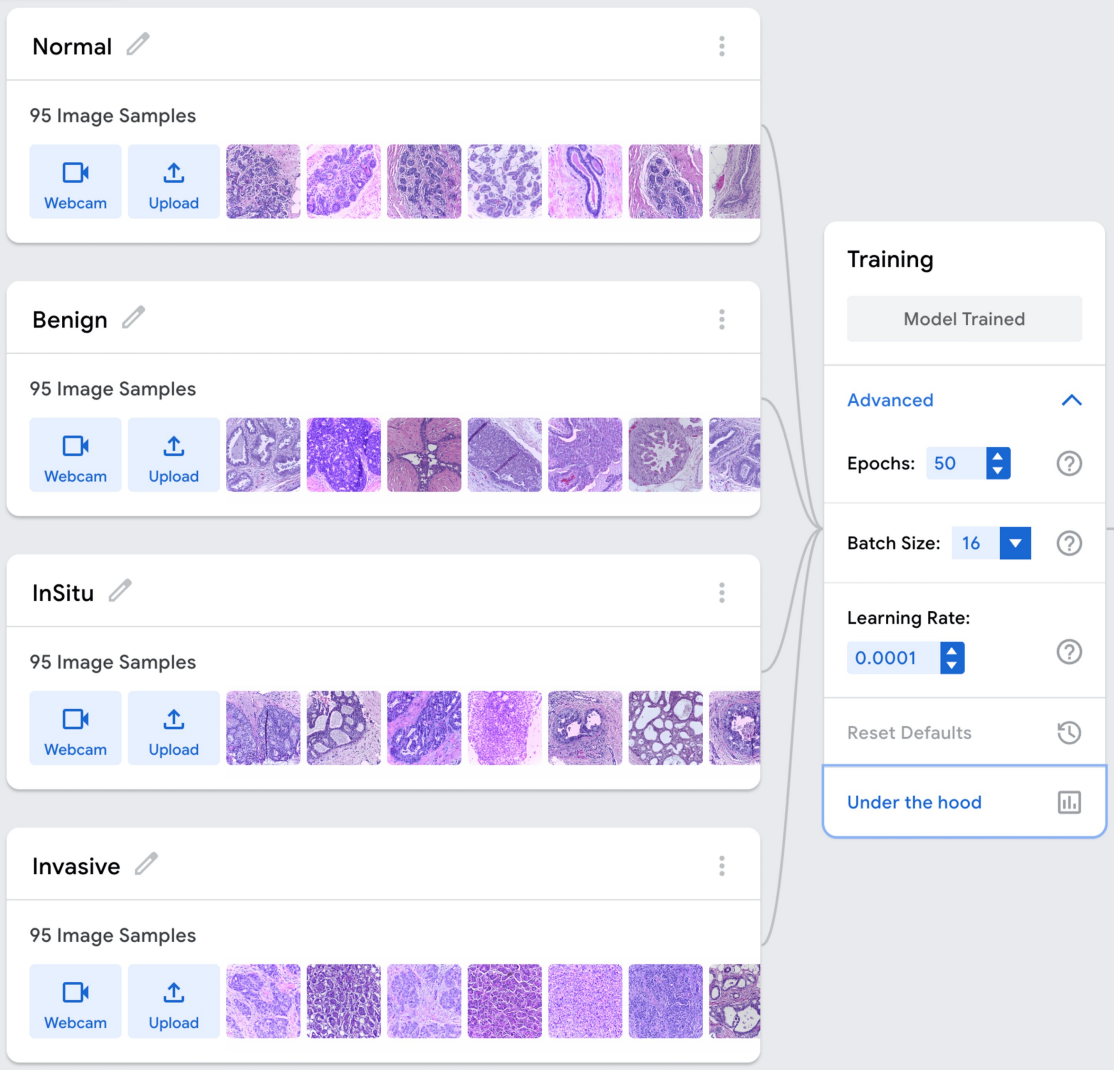
Dataset Composition and Training Parameters for Breast Cancer Histopathological Image Classification Using Google Teachable Machine The dataset included 95 histopathological image samples for each diagnostic category: Normal, Benign, In Situ Carcinoma, and Invasive Carcinoma. The model was trained using 50 epochs, batch size of 16, and learning rate of 0.0001. Images were automatically resized and normalized by the platform prior to training. No formal hypothesis testing was performed due to limited sample size; conventionally, p < 0.05 is considered statistically significant.

Model training was performed using GTM’s advanced settings interface, allowing manual configuration of hyperparameters. The following hyperparameters were selected based on platform recommendations and initial empirical testing for stable convergence: Number of Epochs: 50; Batch Size: 16; Learning Rate: 0.0001 (Figure 1).

The choice of hyperparameters, including a learning rate of 0.0001 and 50 training epochs, was determined after preliminary exploratory runs that evaluated alternative configurations. Adjustments such as higher learning rates or fewer/more epochs were associated with suboptimal model performance, including decreased accuracy and divergent loss curves during training, which indicated instability or overfitting. The selected hyperparameters consistently yielded the best convergence behavior and balanced model performance across internal and external validation sets. These choices were therefore retained as optimal within the constraints of the dataset and platform for this feasibility study. Future studies employing systematic hyperparameter tuning or automated optimization (e.g., grid search or Bayesian optimization) may help identify settings that further enhance classification accuracy and generalizability beyond what was observed in this feasibility study.

The GTM platform employs transfer learning by fine-tuning pre-trained CNNs, such as MobileNet, on the user-supplied dataset. Model optimization was performed using the Adam optimizer. GTM provides real-time visualization of training and validation accuracy and loss curves, which were monitored throughout training to assess convergence behavior and potential overfitting.

Due to platform constraints, no cross-validation, data augmentation, or regularization techniques such as dropout were implemented in this initial feasibility study. The absence of these measures may influence the generalizability and robustness of the trained model, which is addressed in the study limitations.

External model deployment and independent performance evaluation

To objectively assess the model’s generalizability, an independent external validation dataset was created. This external dataset included 39 additional H&E-stained histopathology images, with 10 images each for Normal, Benign, and In Situ Carcinoma classes, and nine images for Invasive Carcinoma. These images were sourced independently from publicly available repositories and local archives, ensuring no overlap with the training dataset. All external images were independently reviewed and verified by expert pathologists using identical diagnostic criteria applied during model training.

The trained GTM model was exported in Keras (.h5) format for external deployment on Google Colaboratory (Colab) [28], utilizing TensorFlow and Keras libraries. The external dataset underwent identical preprocessing steps as applied during model training, including resizing, normalization, and color channel adjustments, to maintain input consistency.

Predictions on the external validation set were generated using Keras model.predict() function. Model performance was evaluated by comparing predicted class labels with true labels, generating multi-class confusion matrices. Performance metrics, including overall accuracy, class-wise precision, recall, and F1 scores, were computed using the scikit-learn classification_report() function.

In addition to class-wise metrics, macro-averaged and weighted-averaged metrics were calculated to account for class imbalance within the external dataset. As this was a preliminary pilot study with limited sample size, no formal statistical hypothesis testing (e.g., McNemar’s test or chi-square tests) was performed; however, confidence intervals for overall performance metrics are reported descriptively.

## Results

Model training performance

**Figure 2:**
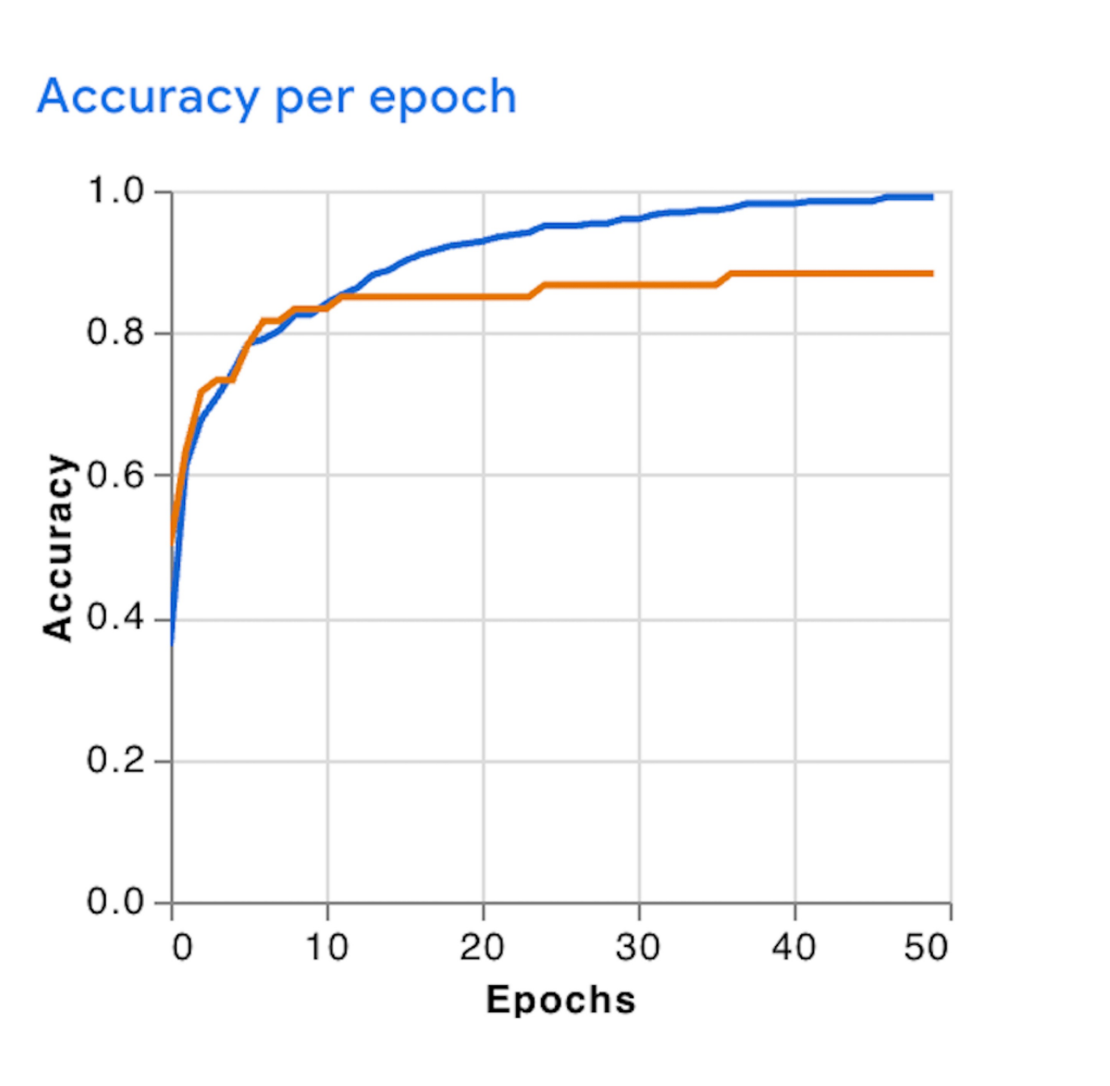
Accuracy Curves During Model Training and Validation Over 50 Epochs The blue line represents training accuracy, and the orange line represents validation accuracy. Both accuracies improved steadily across epochs, with validation accuracy plateauing around 88%.

The GTM model was trained for 50 epochs using a batch size of 16 and a learning rate of 0.0001. During the training phase, both training and validation accuracy improved progressively across epochs. The model achieved near-complete convergence on the training dataset, approaching 100% accuracy. Concurrently, the validation accuracy plateaued at approximately 88% in the final epochs, indicating stabilization during training without significant fluctuations (Figure 2).

**Figure 3:**
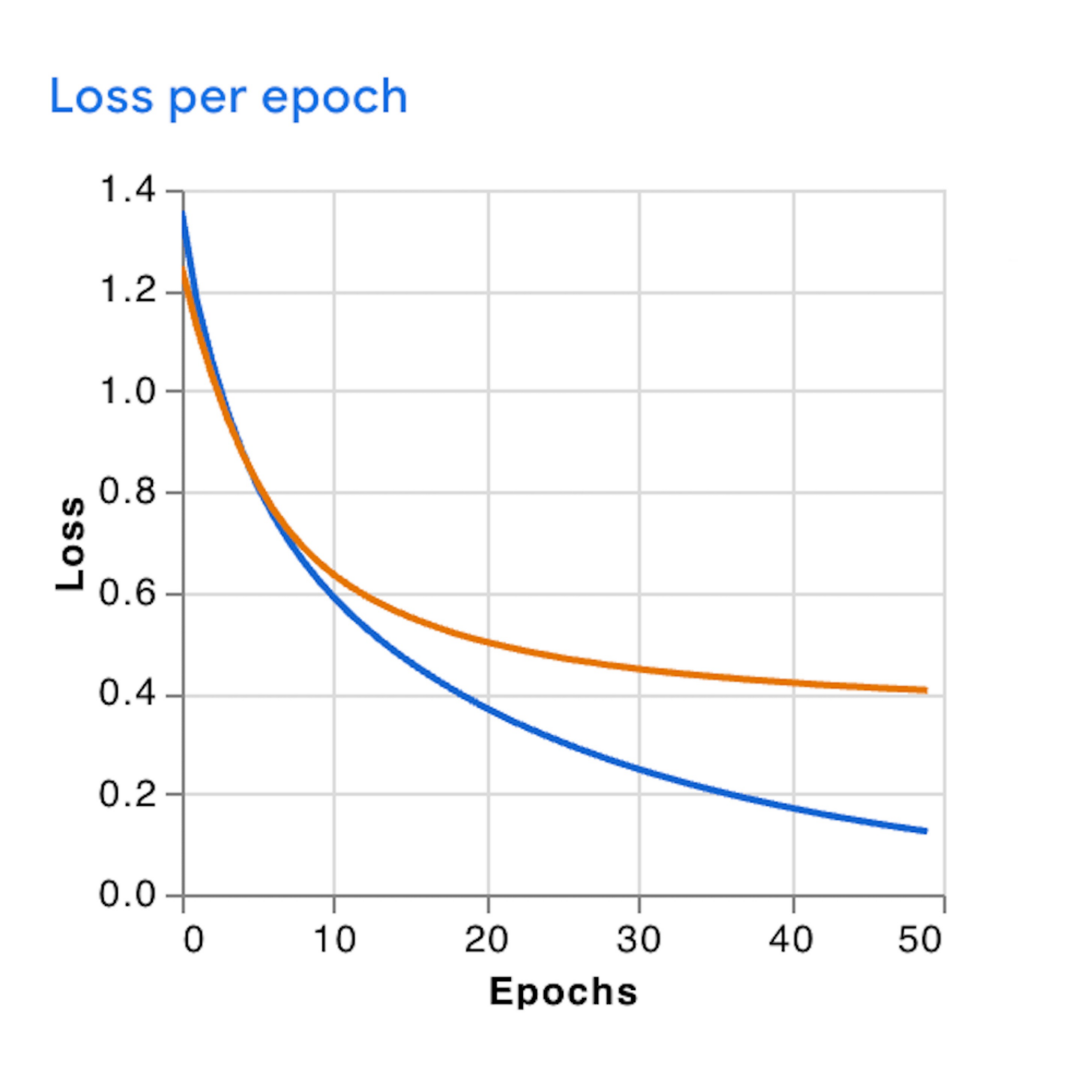
Training and Validation Loss Curves Showing Progressive Reduction in Error Over 50 Epochs The blue line represents training loss, and the orange line represents validation loss. Both loss values decreased progressively across epochs, with validation loss plateauing after approximately 20 epochs.

Training and validation loss curves exhibited smooth, monotonic declines across epochs, suggesting stable convergence behavior without signs of early overfitting (Figure 3). However, the discrepancy between nearly perfect training accuracy and lower validation accuracy indicated the possibility of mild overfitting, likely attributable to the limited dataset size and the absence of augmentation or regularization during model development.

Internal validation performance (GTM 15% split)

GTM automatically allocated 15% of the dataset (60 out of 380 images) for internal testing, with 15 images per diagnostic category. The model demonstrated high per-class accuracies on the internal test set, as shown in Table 1. The overall internal validation accuracy was calculated at 88.3%, with an approximate 95% confidence interval ranging from 77.4% to 95.2%.

**Table 1:** Class-Wise Accuracy of the Model on Internal Validation Dataset (Google Teachable Machine 15% Split) Accuracy values are presented as unitless proportions ranging from 0 to 1, representing the proportion of correctly classified images for each class out of total class samples in the internal validation set (N=15 per class). No formal statistical significance testing was performed due to small sample size; conventionally, p < 0.05 is considered statistically significant.

Class	Accuracy	Samples
Normal	0.87	15
Benign	0.93	15
InSitu	0.87	15
Invasive	0.87	15

**Figure 4:**
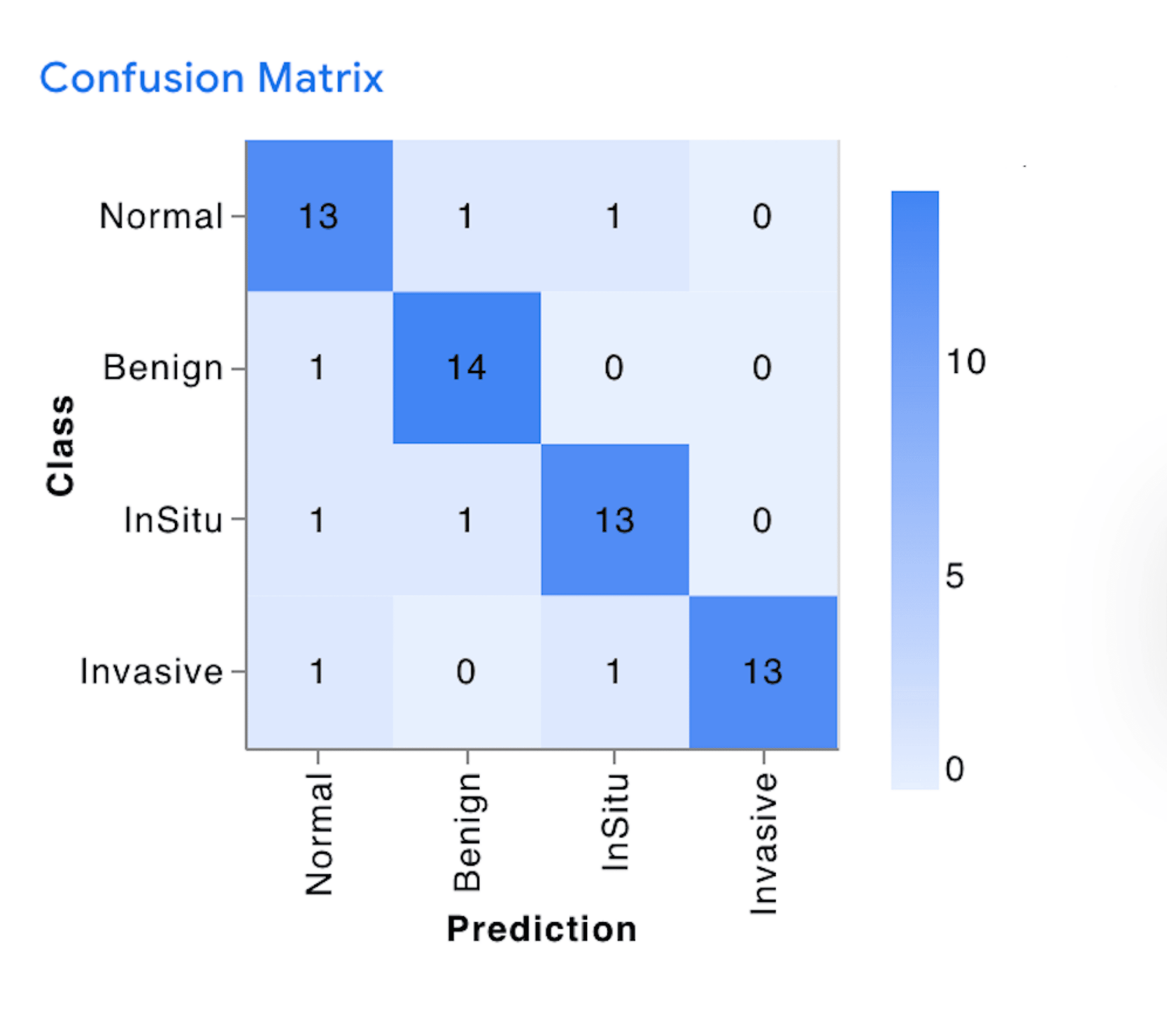
Confusion Matrix for Google Teachable Machine Internal Test Results Showing Correct Identification in Most Classes With Minor Off-Class Predictions The confusion matrix displays the number of samples classified for each true versus predicted class in the internal validation dataset (N = 15 per class). Values inside each cell represent the count of images (unit: number of samples). Diagonal cells indicate correct classifications; off-diagonal cells indicate misclassifications. The color scale corresponds to sample counts, with darker shades representing higher counts (range: 0–15). No formal hypothesis testing was performed due to limited sample size; conventionally, p < 0.05 is considered statistically significant.

Most misclassifications occurred between in situ carcinoma and invasive carcinoma, reflecting overlap in morphological features even at the internal testing stage. Nevertheless, intra-class consistency remained robust across most categories (Figure 4).

External validation performance

The external validation dataset, entirely independent of the training set, comprised 39 histopathology images, distributed as follows: Normal (n=10), Benign (n=10), In Situ Carcinoma (n=10), and Invasive Carcinoma (n=9). The external validation performance metrics demonstrated variability across classes and are provided in Table 2.

**Table 2:** Class-Wise Performance Metrics (Precision, Recall, F1 Score, and Support) for External Validation Dataset F1 scores, recall scores, and precision scores are presented for each diagnostic class as unitless proportions ranging from 0 to 1. Support indicates the number (N) of samples analyzed per class. No formal hypothesis testing was performed due to limited sample size; conventionally, p < 0.05 is considered statistically significant.

Class	Precision	Recall	F1 Score	Support
Normal	1	0.7	0.82	10
Benign	0.67	0.8	0.73	10
In Situ	0.67	1	0.8	10
Invasive	1	0.56	0.71	9

**Figure 5:**
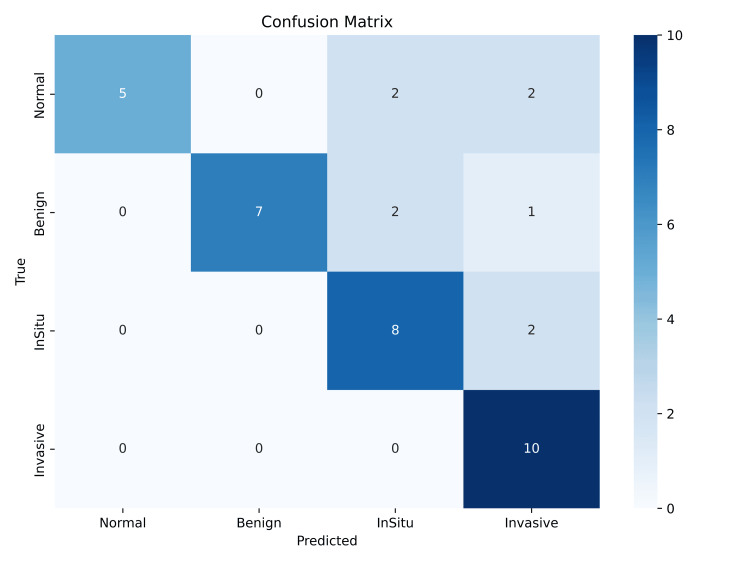
Confusion Matrix of External Validation Dataset The confusion matrix displays the number of samples classified for each true versus predicted class in the external validation dataset (Normal: n=10, Benign: n=10, In Situ Carcinoma: n=10, Invasive Carcinoma: n=9). Values inside each cell represent the count of images (unit: number of samples). Diagonal cells indicate correct classifications; off-diagonal cells indicate misclassifications. The color scale corresponds to sample counts, with darker shades representing higher counts (range: 0–10). No formal hypothesis testing was performed due to limited sample size; conventionally, p < 0.05 is considered statistically significant.

The overall external validation accuracy was 76.9% (95% CI approximately 60.7%-89.0%), with a macro-averaged F1 score of 0.77 and a weighted-averaged F1 score of 0.77. In external validation, the confusion matrix demonstrated correct classification of all invasive carcinoma samples (10/10), while normal, benign, and in situ carcinoma classes showed varying degrees of misclassification. Normal cases exhibited misclassification into in situ and invasive categories. Benign and in situ classes showed minor confusion with neighboring classes (Figure 5).

Although the model exhibited high precision for both the Normal and Invasive Carcinoma classes, its recall for invasive carcinoma dropped to 56%. This lower sensitivity indicated that several invasive carcinoma cases were misclassified into non-invasive categories such as in situ carcinoma, potentially reflecting challenges in differentiating subtle histologic transitions in limited datasets. 

To quantify the degree of overfitting observed during model training, we examined the difference between the final training loss and validation loss at epoch 50. The final training loss was approximately 0.15, while the validation loss stabilized at approximately 0.40. This resulted in a loss delta of 0.25. This divergence between training and validation loss reflects mild overfitting, likely attributable to the small dataset size, absence of data augmentation, and lack of regularization techniques in this preliminary feasibility study.

**Figure 6:**
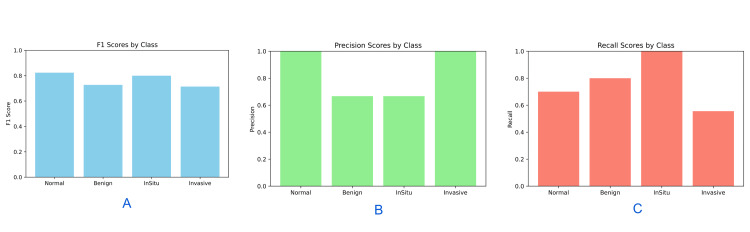
Class-Wise Performance Metrics (bar diagrams) on External Validation Dataset: (A) F1 Score, (B) Precision, and (C) Recall F1 scores, precision scores, and recall scores are presented as unitless proportions ranging from 0 to 1 for each diagnostic class. Precision = True Positives / (True Positives + False Positives); Recall (Sensitivity) = True Positives / (True Positives + False Negatives); F1 Score = Harmonic mean of precision and recall. No formal hypothesis testing was performed due to limited sample size; conventionally, p < 0.05 is considered statistically significant.

Additionally, some Normal images were misclassified into atypical categories, indicating potential false positives for disease presence. Bar diagrams representing F1 score, recall, and precision for each class further highlighted the variability in model performance across diagnostic categories (Figure 6).

Overfitting considerations

Despite achieving near-perfect training accuracy, the internal test accuracy plateaued below 90%, and the external validation accuracy declined further to approximately 77%. This performance gap, coupled with limited sample size and absence of data augmentation or regularization, suggests the presence of mild overfitting. Incorporating additional data, augmentation, and regularization techniques may help mitigate this effect in future model iterations.

Statistical testing

Given the pilot nature of this study and the limited sample size, no formal statistical hypothesis testing (e.g., McNemar’s test or chi-square tests) was conducted. Instead, confidence intervals were reported to provide an estimate of variability in observed accuracies. Larger datasets will be necessary to enable robust statistical comparisons and hypothesis-driven evaluations.

Table 3 summarizes the performance across various evaluation phases. The training epochs achieved over 98% accuracy, demonstrating stable convergence but also indicating potential overfitting. Internal GTM testing results are pending further elaboration.

**Table 3:** Summary of Performance Across Evaluation Phases GTM: Google Teachable Machine, Overall accuracy is presented as proportion (%). Strengths and limitations summarize key observations for each evaluation phase. No formal hypothesis testing was performed due to limited sample size; conventionally, p < 0.05 is considered statistically significant.

Evaluation Phase	Overall Accuracy	Strengths	Limitations
Training Epochs	>98%	Stable convergence	Potential overfitting
Internal GTM Test (15%)	88.3%	High accuracy across classes	Some in situ/invasive misclassifications
External Validation	76.9%	Maintains multi-class separation	Lower recall for invasive carcinoma

## Discussion

The integration of AI and ML into breast cancer diagnostics has shown substantial promise for enhancing accuracy, reproducibility, and efficiency in digital pathology workflows. In this pilot study, a no-code, browser-based AI platform, GTM, was evaluated for its ability to classify breast histopathology images into four clinically relevant categories. The study demonstrated encouraging internal performance (88.3% accuracy) and moderate generalizability on external validation (76.9% accuracy), suggesting preliminary feasibility of such platforms for histopathological classification tasks.

The performance observed in this study aligns with accumulating evidence that DL and CNNs can achieve high diagnostic accuracy in histopathological tasks such as tumor detection, mitotic figure counting, and tumor grading [3,9]. Importantly, the consistent intra-class accuracy seen during internal validation suggests that even with limited computational resources and minimal technical expertise, no-code platforms like GTM can generate functional classification models.

However, several important limitations emerged during external validation, particularly in the classification of invasive carcinoma, where recall dropped to 56%. This reduced sensitivity indicates that the model misclassified multiple invasive carcinoma cases into non-invasive categories such as in situ carcinoma. This observation mirrors the diagnostic complexity faced by human pathologists, as histological features between in situ and invasive carcinoma can be morphologically overlapping and challenging to distinguish [6]. From a clinical perspective, such under-diagnosis of invasive carcinoma is significant, as it may directly influence patient management and therapeutic decisions.

The imbalance in class-wise recall highlights a fundamental issue related to limited training data diversity. Unlike high-volume, fully supervised models that are trained on tens of thousands of images, this study employed a relatively small dataset comprising 380 images for training and 39 images for external validation. The absence of data augmentation or regularization during training likely further restricted the model’s exposure to the spectrum of histopathological variability, contributing to its limited generalization performance.

In addition to dataset size, the lack of hyperparameter tuning, advanced regularization methods, and cross-validation also constrained the model’s robustness. While GTM allows rapid model development through simplified interfaces, it provides minimal flexibility for architectural customization, hyperparameter optimization, or implementation of complex training pipelines. Such limitations, while acceptable for initial feasibility assessments, become increasingly relevant when considering translation into clinical practice.

Another important consideration is the interpretability of deep learning models. The present study did not incorporate explainability frameworks such as saliency mapping, Grad-CAM, or attention-based visualizations, which can help elucidate the morphological regions contributing to model predictions. The “black box” nature of such models remains a critical barrier for clinical trust and regulatory acceptance [12]. Future iterations should integrate explainable AI (XAI) methodologies to enhance transparency, interpretability, and clinical adoption.

The accessibility of platforms like GTM represents one of their primary advantages, particularly for low-resource settings where access to expert pathologists and computational infrastructure is limited. Democratizing AI development through no-code solutions may facilitate broader deployment of AI-assisted diagnostic tools in regions that face workforce shortages and increasing diagnostic demands [11]. However, clinical deployment would require rigorous external validation on large, diverse, and multicenter datasets, along with careful performance benchmarking against expert pathologists.

Beyond diagnostic classification, AI-driven histopathological analysis holds additional promise for prognostication and treatment stratification. Quantification of tumor-infiltrating lymphocytes (TILs), mitotic activity, and molecular correlates may provide critical information on patient outcomes and therapeutic responsiveness [26]. Incorporating multimodal data streams could further enhance model performance, particularly for complex classifications such as invasive carcinoma differentiation.

Despite these future opportunities, it remains critical to recognize the current barriers to the real-world integration of AI models in pathology. Regulatory approval processes, standardized reporting guidelines, algorithmic validation protocols, and data privacy considerations will all play essential roles in ensuring patient safety and fostering clinical adoption [10].

In summary, this study demonstrated that no-code AI platforms like Google Teachable Machine can serve as feasible tools for initial model development in breast histopathology image classification. The model’s reasonable internal performance and partial external generalization highlight both its potential and its current limitations. Future research should prioritize larger datasets, advanced training architectures, robust cross-validation frameworks, incorporation of explainable AI techniques, and comprehensive clinical validation to optimize model performance and facilitate safe clinical translation.

Limitations

This study has several important limitations. First, the dataset size was small for a multi-class classification task, which restricted the model’s generalizability and contributed to mild overfitting. Second, the model was developed without data augmentation, regularization, or systematic hyperparameter optimization, which likely limited its robustness and performance. Third, although confidence intervals were provided, no formal statistical significance testing was conducted due to the limited sample size. Finally, while Google Teachable Machine offers valuable accessibility for non-programmers and resource-constrained settings, its black box nature limits control over model architecture, hyperparameter tuning, and output explainability - factors that are essential for clinical trust, interpretability, and regulatory approval. Nevertheless, for initial feasibility studies, GTM remains a useful platform for exploring the integration of AI into digital pathology workflows.

## Conclusions

In this pilot study, the feasibility of applying a no-code machine learning platform, Google Teachable Machine, for multi-class classification of breast histopathology images was evaluated. The model achieved reasonable internal accuracy and demonstrated partial generalization on an independent external dataset, despite the small sample size and absence of advanced optimization techniques. Importantly, while the model preserved class separation in most categories, reduced recall for invasive carcinoma highlighted persistent diagnostic challenges inherent to both AI systems and human pathologists, particularly when differentiating between invasive and non-invasive lesions.

The findings emphasize the potential role of no-code AI platforms as accessible tools for educational purposes, research prototyping, or preliminary diagnostic support, especially in resource-limited environments where specialist expertise and computational resources may be scarce. However, the observed limitations also underscore the necessity for larger, more diverse datasets, advanced training architectures, and incorporation of explainable AI methods to improve model robustness, transparency, and clinical applicability. Further prospective multicenter studies with rigorous external validation will be essential to advance such AI systems toward safe clinical integration.
